# *IKZF3* amplification predicts worse prognosis especially in intestinal-type gastric cancer

**DOI:** 10.1007/s00432-024-05868-2

**Published:** 2024-07-25

**Authors:** Zhaomeng Cui, Huaiyu Liang, Rongkui Luo, Wen Huang, Wei Yuan, Lei Zhang, Lijuan Luan, Jieakesu Su, Jie Huang, Chen Xu, Yingyong Hou

**Affiliations:** 1 Department of Pathology, Zhongshan Hospital, Fudan University, Shanghai, 200032 China; 2 Department of Pathology, Xiamen Branch of Zhongshan Hospital, Fudan University, Xiamen, 361015 Fujian China

**Keywords:** *IKZF3*, *HER2*, Gastric cancer, Intestinal-type, Prognosis

## Abstract

**Purpose:**

IKAROS family zinc finger 3 (*IKZF3*) is an oncogene involved in different malignancies, particularly in the development and malignant progression of lymphocytes. However, *IKZF3* amplification and clinical significance in gastric cancers (GCs) remain unexplored.

**Methods:**

We examined *IKZF3* amplification status in 404 GCs with *HER2* amplification status using tissue microarray (TMA) and fluorescence in situ hybridization (FISH) assays.

**Results:**

*IKZF3* amplification was detected in 6.9% (28/404) of all GC patients, with higher rates in intestinal-type gastric cancer (IGC) (11.22%, 22/196) compared to other types (2.88%, 6/208). *HER2* amplification was identified in 16.09% (65/404) of all GC patients, with higher rates in IGC (20.92%, 41/196) compared to other types (11.54%, 24/208). Co-amplification of *IKZF3* and *HER2* was detected in 8.16% (16/196) of IGC patients and in 2.40% (5/208) of other types. *IKZF3* amplification showed significant correlation with IGC (*P* = 0.001) and *HER2* amplification (*P* = 0.0001). *IKZF3* amplification exhibited significantly worse disease-free survival (DFS) (*P* = 0.014) and overall survival (OS) (*P* = 0.018) in GC patients, particularly in IGC (DFS: *P* < 0.001; OS: *P* < 0.001), rather than other types. Cox regression analysis demonstrate *IKZF3* amplification as an independent poor prognostic factor in all GCs (*P* = 0.006, *P* = 0.004 respectively) and in IGC patients, regardless of stages I-II or III-IV (*P* = 0.007, *P* = 0.004 respectively). On the other hand, *HER2* amplification was significantly associated with worse DFS (*P* = 0.008) and OS (*P* = 0.01) in IGC patients, but not in all GCs and in multivariate analysis. Within the subset of patients with *HER2* amplification, those also exhibiting *IKZF3* amplification displayed potential poorer prognosis (*P* = 0.08, *P* = 0.11 respectively).

**Conclusion:**

*IKZF3* amplification was detected in minority of GC patients, especially in IGC, and was an independent indicator of poor prognosis. Our study, for the first time, found the prognostic value of *IKZF3* was superior to *HER2* for GC patients.

**Supplementary Information:**

The online version contains supplementary material available at 10.1007/s00432-024-05868-2.

## Introduction

Gastric cancer, ranking fifth in terms of occurrence and second in global mortality caused by cancer, exhibits heterogeneous behaviors at multiple levels, including genetic mutations, tumor microenvironment, and cellular characteristics (Smyth et al. 2020). Approximately 50% of GC cases exist in east Asia (Liu et al. 2019). Various classifications were proposed to evaluate the histopathology of GC. Lauren’s classification system was proposed since 1965 (Lauren 1965), which is useful and widely used in GC to date. Generally, GC is classified into intestinal-type, diffuse-type and mixed-type (Aravind Sanjeevaiah 2018). Genetic alteration in different histologic types has been studied widely because of distinct pathological types, epidemiology, epigenetic aberrations and prognosis (Chia 2016). To date, surgical resection remains the major therapeutic strategy for current management of GC. Even though, the overall outcomes of GC still remain dismal because of the aggressive cancer behaviors and high recurrent rate (Chen et al. 2015; Song et al. 2017). The survival time of advanced GC patients is less than 12 months generally, and 5-year survival rate is less than 10% (Song et al. 2017). Besides, GC patients still bear huge medical costs, and there remains a great unmet need for treatment options to achieve better clinical outcomes.

In general, gastric cancer is highly heterogeneous in both clinical behaviors and molecular levels, our understandings of the changes of genetic landscape remains limited. Since the advent of targeted therapy, a game-changer in oncologic management in GC patients with *HER2* amplification, for example, trastuzumab, many other related researches to explore the significance of gene amplification in GC rapidly intensified. Gene amplification generally refers to an increase in the number of gene copies in a specific region of a chromosome (Albertson 2006), exerting many biological effects on genome stability, cell proliferation, differentiation, invasion, metastasis, apoptosis and angiogenesis (Douglas H 2000), as well as being involved in drug resistance. Several amplified genes including *ERBB2* (Kanayama et al. 2018; Nakata et al. 2019; Ughetto et al. 2021), *FGFR2* (Kunii et al. 2008), *MET* (Hou et al. 2019), *TNK2* (Shinmura et al. 2014) have been identified significantly associated with advanced tumor grade and heightened aggressiveness in gastric cancer. The investigation into the therapeutic potential of amplification in other oncogenes has been actively pursued in recent years. Even though, the understanding of numerous common amplifications in GC remains limited, which might be beneficial for oncology studies and development of related precision medicines (Lin et al. 2022). In our study, we investigated common gene amplification events in GC, which might be of clinical interest. We queried stomach cancer databases (TCGA, Nature 2014) deposited in cBioPortal (http://cbioportal.org), and the frequency of all amplified genes were ranked. An arbitrary cut-off of 10% frequency for gene amplification was used and *IKZF3* was selected for further analysis in 14 genes, with the amplification frequency 11.3%. Our study confirmed *IKZF3* amplification is commonly occurred in GC. *IKZF3* belongs to the Ikaros family of zinc-finger proteins, which play an essential role in lymphatic differentiation, development and maintenance of homeostasis. There are five homologous members within the Ikaros transcription factor family, namely Ikaros (*IKZF1*), Helios (*IKZF2*), Aiolos (*IKZF3*), Eos (*IKZF4*), and Pegasus (*IKZF5*) (Heizmann et al. 2018).

*IKZF3* expression in T-cells was reported as a predictive indicator for clinical outcomes in multiple myeloma patients (Awwad et al. 2018). Other researches showed mutant *IKZF3* drives B cell neoplasia by modulating BCR and NF-κB signaling (Lazarian et al. 2021). Besides, it had been shown that high expression of *IKZF3* usually indicate positive immunological responses and beneficial clinical results of skin cutaneous melanoma (Yang et al. 2022). *IKZF3* has also been reported to have regulatory role in breast cancer, head and neck cancers, auto-immune diseases, and brain ischemia-reperfusion injury (Li et al. 2018, 2023; Lin et al. 2022; Meng et al. 2023). Very few studies have reported the significance of *IKZF3* in GC. Here we investigate the potential clinical significance of *IKZF3* in GC. Our data suggest that *IKZF3* amplification often occurs in GC, and is predominantly altered in IGC. Our results revealed *IKZF3* as a promising target in GC for both academic investigations and therapeutic development.

## Methods

### Patients selection

This study comprised a cohort of 404 patients who underwent surgical procedures for stomach lesions and were diagnosed with gastric cancer in Zhongshan Hospital, Fudan University, between January 2015 and January 2020. Patients who had received preoperative antitumor treatment, such as chemotherapy, neoadjuvant therapy and radiotherapy were excluded from this cohort.

All available hematoxylin and eosin (H&E) stained slides were independently reviewed and assessed by two senior pathologists (Hou YY, Xu C). The diagnostic criteria used for all cases were in accordance with the standard guidelines outlined in the fifth edition of the WHO classification of digestive system tumors, all cases were staged based on the rules specified within the eighth edition cancer staging manual of the American Joint Cancer Committee.

Clinicopathological characteristics including gender, age, lymph node metastasis (LNM), EBV infection, clinical stage and disease progression were obtained from related clinical records. Follow-up data of patients who did not revisit Zhongshan hospital for post-operative check were then interviewed by phone with patients and/or their families. Missed visit were exclude from this study.

Informed consent for the acquisition and use of tissue specimens and related clinical data of GC patients was obtained ahead of time from each patient. The Human Research Ethics Committee of Zhongshan Hospital, Fudan University has granted ethical approval for this study.

### Tissue microarray preparation

The construction of tissue microarrays (TMAs) comprising tumor tissues of 404 GC patients was referred to the previous description (Yuan Shi 2013). In short, the representative regions of 2 × 6 mm with abundant tumor cells were chosen by two senior pathologists based on HE-staining results. The precise area on the formalin-fixed, paraffin-embedded (FFPE) tissue blocks were isolated subsequently, arrayed onto the recipient TMA blocks vertically and consolidated on precision instrument (Leica EG1150).

### Fluorescence in situ hybridization

Dual-color FISH assay using a specific probe for *IKZF3* or *HER2* (Spectrum orange) in combination with a centromere-specific probe (Spectrum green) targeting chromosome 17 (CEP17) (Empire Genomics, Buffalo, NY) was administered on the TMA sections of 3 μm thickness based on standard operation procedure (Oshima et al. 2014) to evaluate *IKZF3* or *HER2* amplification. *IKZF3* or *HER2* amplification was confirmed by two senior pathologists blinded to patients’ clinical features under a fluorescence microscope (BX43; Olympus, Japan) equipped with a DAPI/green/orange triple band pass filter and a microscope digital camera (DP50; Olympus, Japan). *IKZF3, HER2* and CEP17 signals were counted in at least 100 tumor cell nuclei of each GC sample under an oil microscope at 1,000x magnification. Amplification of *IKZF3* was defined according to previously documented scoring algorithms for *HER2* (Balestra et al. 2023). That is, *IKZF3*/CEP17 ratio ≥ 2.0.

### Immunohistochemistry (IHC)

The IHC assay using IKZF3 rabbit monoclonal antibody (Abcam, ab139408) was administered on the TMA sections with the Ventana iView DAB Detection Kit on a BenchMark XT automated staining system (Ventana Medical Systems, Tucson, AZ). Any nuclear expression of IKZF3 is considered positive.

### Statistical analysis

The statistical analysis were administered with GraphPad Prism 9.4.1. All P-values were two sided, a *P* ≤ 0.05 were considered statistically significant. Pearson χ^2^ test was performed to calculate the correlation between *IKZF3* amplification and various clinicopathologic variables in GC patients. The Kaplan–Meier method with log-rank test was utilized to calculate the correlation between *IKZF3/HER2* amplification and the survival ratio of OS and DFS, and to determine whether the survival curves were significantly different. The Cox proportional hazard regression model was administered for both univariate and multivariate analysis, with the hazard ratio (HR) were obtained.

## Results

### Clinical and pathological features

**Fig. 1:**
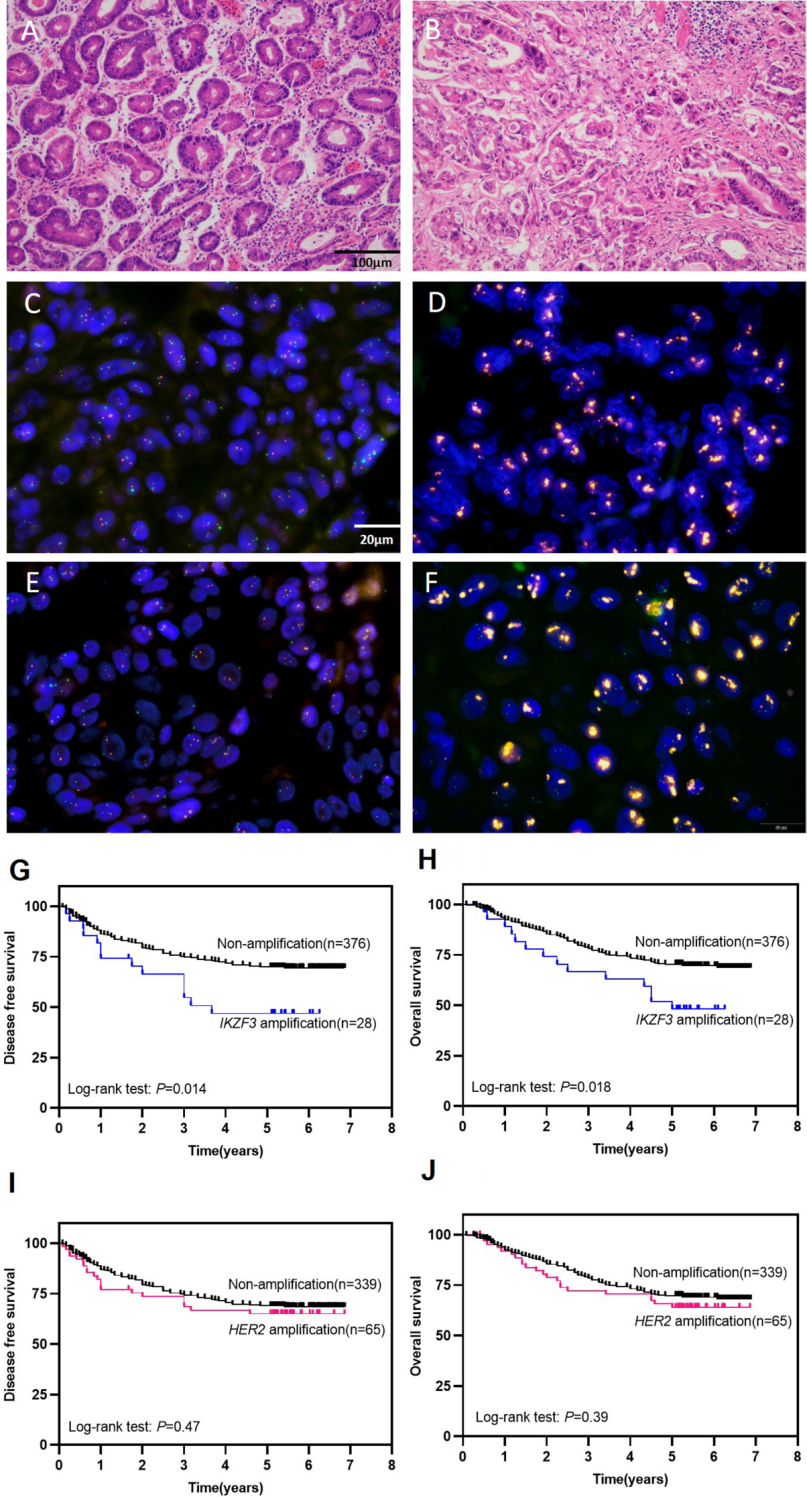
**(A-B)** Representative images of H&E-staining (×200) in GC patients. **A** Intestinal-type GC, **B** Mix-type GC. **(C-F)** Representative patterns of *IKZF3* or *HER2* gene (orange color) and CEP17 (green color) status by FISH (×1,000). **C***IKZF3* non-amplification, **D ***IKZF3* amplification. **E ***HER2* non-amplification, **F***HER2* amplification. (**G-H**) Kaplan–Meier curves of poorer disease-free survival (DFS) and overall survival (OS) of cases with *IKZF3* amplification in 404 GC patients. (**I-J)** Kaplan–Meier curves indicate no difference in DFS and OS between cases with or without *HER2* amplification

We summarized the main clinical and pathological features of the 404 patients with GC (Table 1). The mean age was 63.5 years (overall range 24 to 84 years). Among these GC patients, 298 (73.8%) were male. pTNM stage was determined based on pathologic evaluations, 222 cases (54.9%) were staged I & II and 182 (45.1%) cases were staged III & IV. Vessel invasions were identified in 200 (49.5%) cases, neural invasions in 189 (46.8%) cases. Lymph node metastasis was observed in 61.1% (247 of 404) of GC patients. For histologic subtyping in GC, a further classification was made according to Lauren’s criteria. 196 (48.5%) cases of intestinal-type (Fig. 1A), 179 (44.3%) cases of mixed-type (Fig. 1B), and 22 (5.44%) cases of diffuse-type were identified. During the follow-up period, 37.3% (151 of 404) patients developed disease progression. 23.8% (96 of 404) patients died of GC.

**Table 1:** The correlation between *IKZF3* amplification and clinical characteristics in 404 patients with gastric cancer

Clinicopathologic features	Non-amplification *N* = 376	*IKZF3* amplification *N* = 28	*p*-value
**Sex**			
Male	274	24	0.14
Female	102	4	
**Age (years)**			
< 60	112	6	0.35
≥ 60	264	22	
**pTNM**			
I-II	206	16	0.81
III-IV	170	12	
**Differentiation Grading**			
Well or Moderately	66	4	0.66
Poorly	310	24	
**Lauren classification**			
Intestinal	174	22	0.001^**^
Mixed	174	5	
Diffuse (signet-ring cell carcinoma)	21	1	
Solid	7	0	
***HER2***			
Non-amplification	332	7	0.0001^***^
Amplification	44	21	
**LN metastasis**			
No	148	9	0.45
Yes	228	19	
**EBV infection**			
Negative	354	27	0.62
Positive	22	1	
**Tumor Deposits** ^☆^			
No	305	23	0.89
Yes	71	5	
**Vessel invasion**			
No	189	15	0.74
Yes	187	13	
**Nerve invasion**			
No	198	17	0.41
Yes	178	11	
**Disease progression** ^★^			
No	241	12	0.03^*^
Yes	135	16	
**Cancer-related Death**			
No	290	18	0.12
Yes	86	10	
**LN metastasis(EGC**, ***n*=88)**^†^			
No	61	3	0.91
Yes	23	1	
**Vessel invasion(EGC**, ***n*=88)**			
No	65	1	0.018^*^
Yes	19	3	

^☆^Tumor Deposits is defined as clusters of cancer cells in the soft tissue that are discontinuous from the primary tumor

^★^Disease progression is defined as relapse and metastasis

^†^EGC: early-stage GC patients (Tis + T1a + T1b)

### The relevance between *IKZF3* amplification and clinicopathological characteristics of GC patients

We evaluated *IKZF3* amplification in 404 patients with GC by FISH. *IKZF3* amplification (*IKZF3*/CEP17 ratio ≥ 2.0) was found in 6.9% (28 of 404) of patients (Fig. 1D), and other patients (93.1%, 376 of 404) showed non-amplification (Fig. 1C). The associations between *IKZF3* amplification and related clinicopathological characteristics are presented in Table 1. *IKZF3* amplification status was found significantly correlated with intestinal-type GC (*P* = 0.001), disease progression (*P* = 0.03), and *HER2* amplification (*P* = 0.0001) (Fig. 1E-F). There was no significant difference between *IKZF3* amplification and *IKZF3* wild type group considering sex (*P* = 0.14), age (*P* = 0.35), pTNM stage (*P* = 0.81), differentiation grading (*P* = 0.66), lymph node metastasis (*P* = 0.45), EBV infection (*P* = 0.62), tumor deposits (*P* = 0.89), vessel invasion (*P* = 0.74), nerve invasion (*P* = 0.41), and cancer related death (*P* = 0.12). Given the strong correlation between *IKZF3* amplification and intestinal-type GC, we then analyzed its association with lymph node metastasis and vessel invasion in early-stage GC patients (Tis + T1a + T1b). *IKZF3* amplification was found not related to LNM, but significantly correlated with vessel invasion in early-stage GC patients (Table 1). Additionally, IHC assay was performed on the TMA sections to assess IKZF3 protein expression, with the results that low protein expression of IKZF3 was detected in 12 cases with *IKZF3* amplification, while no IKZF3 expression was detected in the remaining 16 cases with *IKZF3* amplification (Assessed by two senior pathologists) (Supplemental information Fig. 1). This proves the protein expression of IKZF3 does not significantly correlate with amplification status.

### *IKZF3* amplification is associated with poor prognosis in GC patients

Survival data were analyzed of all 404 GC patients. The 5-year DFS and OS rates were 56.7% (229/404) and 58.9% (238/404) respectively, the median follow-up period is 63.66 months (range 1–82.5 months). Mean and median duration of DFS were 48.9 and 62.9 months, while to OS were 52.1 and 64.2 months respectively. To further investigate the prognostic value and clinical outcomes of *IKZF3* amplification in GC, Kaplan–Meier analysis was performed with the results that compared with the group without *IKZF3* amplification (*n* = 376, median DFS, 50.9 months; median OS, 53.8 months), cases with *IKZF3* amplification (*n* = 28) gained significant worse survival with a median DFS 39.7 months (*P* = 0.014) and median OS 45.7 months (*P* = 0.018) respectively (Fig. 1G-H). As a comparison, the survival data of all GC patients with *HER2* amplification were also analyzed, and the results showed no significant difference between GC patients with or without *HER2* amplification in terms of both DFS (*P* = 0.47) and OS (*P* = 0.39) (Fig. 1I-J). Besides, univariate analysis of prognostic significance suggests that age, pTNM stage, Lauren classification, LN metastasis, *IKZF3* amplification, tumor deposits, vessel invasion and nerve invasion were significantly associated with DFS and OS in all GC patients (Table 2). In the multivariate analysis, age (*P* = 0.002, HR: 0.485 for DFS; *P* = 0.001, HR: 0.466 for OS), pTNM stage (*P* = 0.032, HR: 1.806 for DFS; *P* = 0.023, HR: 1.868 for OS), LN metastasis (*P* = 0.029, HR: 2.246 for DFS; *P* = 0.035, HR: 2.184 for OS), *IKZF3* amplification (*P* = 0.006, HR: 2.286 for DFS; *P* = 0.004, HR: 2.416 for OS), tumor deposits (*P* < 0.001, HR: 1.322 for DFS; *P* < 0.001, HR: 1.300 for OS), vessel invasion (*P* = 0.04, HR: 1.611 for DFS; *P* = 0.03, HR: 1.655 for OS), and nerve invasion (*P* = 0.021, HR: 1.678 for DFS; *P* = 0.012, HR: 1.750 for OS) were associated with DFS and OS (Table 2). These findings imply that *IKZF3* amplification stands as an independent prognostic factor across all 404 GC cases.

**Table 2:** Univariate and multivariate survival analysis for OS and DFS in all GC patients and in IGC patients

Variable	DFS(GC)		OS(GC)		DFS (IGC)		OS(IGC)	
	HR	*P* value	HR	*P* value	HR	*P* value	HR	*P* value
**Univariate analysis**								
Sex	0.912	0.660	0.897	0.607	0.429	0.073	0.435	0.078
**Age (years)**	0.487	0.002	0.483	0.002	0.530	0.072	0.511	0.044
**pTNM stage**	4.864	<0.001	4.842	<0.001	4.546	<0.001	4.744	<0.001
**Lauren classification**	0.554	0.001	0.564	0.003				
***HER2 *** **amplification**	1.456	0.100	1.430	0.117	2.219	0.007	2.150	0.010
**LN metastasis**	6.483	<0.001	6.488	<0.001	5.522	<0.001	5.683	<0.001
***IKZF3 *** **amplification**	1.946	0.020	1.954	0.019	3.718	<0.001	3.706	<0.001
EBV infection	0.258	0.178	0.340	0.283	0	0.999	0	0.999
**Tumor deposits**	1.517	<0.001	1.487	<0.001	1.478	<0.001	1.438	<0.001
**Vessel invasion**	3.488	<0.001	3.516	<0.001	2.522	0.001	2.555	0.008
**Nerve invasion**	3.381	<0.001	3.498	<0.001	3.323	<0.001	3.405	<0.001
**Multivariate analysis**								
**Age (years)**	0.485	0.002	0.466	0.001				
**pTNM stage**	1.806	0.032	1.868	0.023	1.729	0.160	1.992	0.068
Lauren classification	0.894	0.594	0.923	0.700				
*HER2* amplification					1.140	0.698	1.032	0.927
**LN metastasis**	2.246	0.029	2.184	0.035	2.284	0.071	2.290	0.063
***IKZF3*** **amplification**	2.286	0.006	2.416	0.004	3.381	0.007	3.734	0.004
**Tumor deposits**	1.322	<0.001	1.300	<0.001	1.334	<0.001	2.519	0.003
**Vessel invasion**	1.611	0.040	1.655	0.030	1.112	0.744	1.135	0.699
**Nerve invasion**	1.678	0.021	1.750	0.012	1.673	0.097	1.689	0.091

**Fig. 2:**
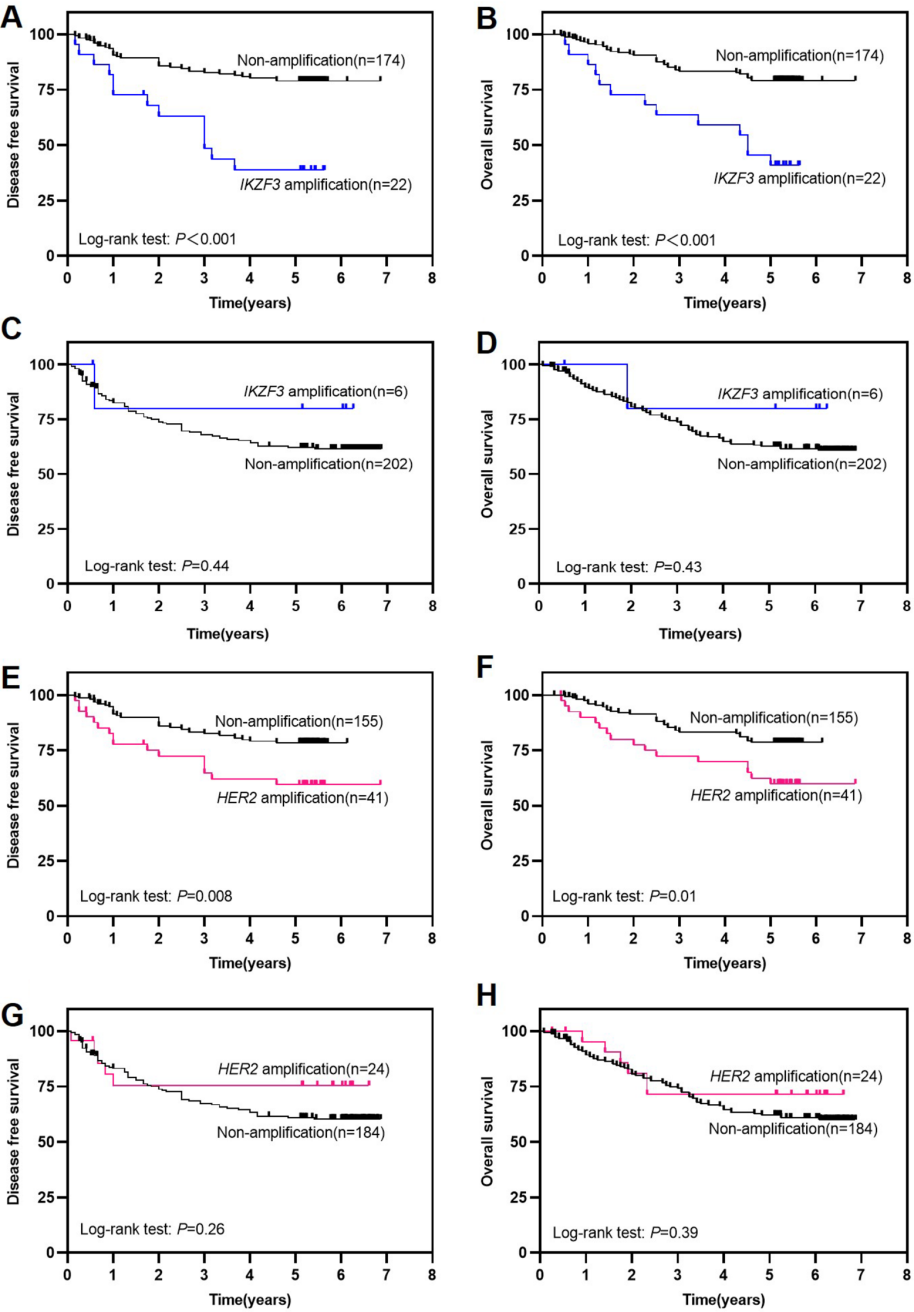
Survival analyses based on Lauren classification of GC patients. **(A, B) ***IKZF3* amplification was associated to poorer DFS and OS in IGC patients. **(C, D) ***IKZF3* amplification was not corelated with DFS and OS in other types GC patients. **(E, F) ***HER2* amplification was linked to poorer DFS and OS in IGC patients. **(G, H) ***HER2* amplification was not associated to DFS and OS in other types GC patients

In our study, twenty-two out of 28 patients with *IKZF3* amplification were histologically classified as IGC referring to Lauren classification. Therefore, we administered relevant survival analysis among GC patients from different Lauren subtypes. In IGC patients (*n* = 196, Fig. 2A-B), *IKZF3* amplification was discovered to exhibit a substantial correlation with unfavorable outcomes, with a median DFS and OS being 37.1 and 44.0 months compared with 52.8 and 54.9 months for 174 patients without *IKZF3* amplification (*P* < 0.001 for DFS and *P* < 0.001 for OS). In other types GC patients (*n* = 208, Fig. 2C-D), *IKZF3* amplification did not have a significant impact on DFS (*P* = 0.44) or OS (*P* = 0.43). The median duration of DFS and OS for 6 patients with *IKZF3* amplification was 42.3 and 48.5 months respectively, compared to 49.6 and 53.0 months for the remaining 202 patients without *IKZF3* amplification. It is worth pointing out that in IGC, patients with *HER2* amplification (*n* = 41) have poorer DFS (*P* = 0.008) and OS (*P* = 0.01) compared to those without *HER2* amplification (*n* = 155, Fig. 2E-F). However, in other types of GC patients, *HER2* amplification is not significantly correlated with patient prognosis (*P* = 0.26 for DFS, *P* = 0.39 for OS) (*n* = 208, Fig. 2G-H). Univariate analysis suggests that pTNM stage, *HER2* amplification, LN metastasis, *IKZF3* amplification, tumor deposits, vessel invasion, and nerve invasion were significantly linked to DFS and OS in IGC patients (Table 2). In the multivariate analysis, *IKZF3* amplification (*P* = 0.007, HR: 3.381 for DFS; *P* = 0.004, HR: 3.734 for OS) and tumor deposits (*P* < 0.001, HR: 1.334 for DFS; *P* = 0.003, HR: 2.519 for OS) were associated with DFS and OS (Table 2). These data demonstrate that *IKZF3* amplification holds an independent prognostic value for survival in IGC patients. Meanwhile *HER2* amplification is linked to unfavorable prognosis in IGC patients, whereas not an independent prognostic factor.

**Fig. 3:**
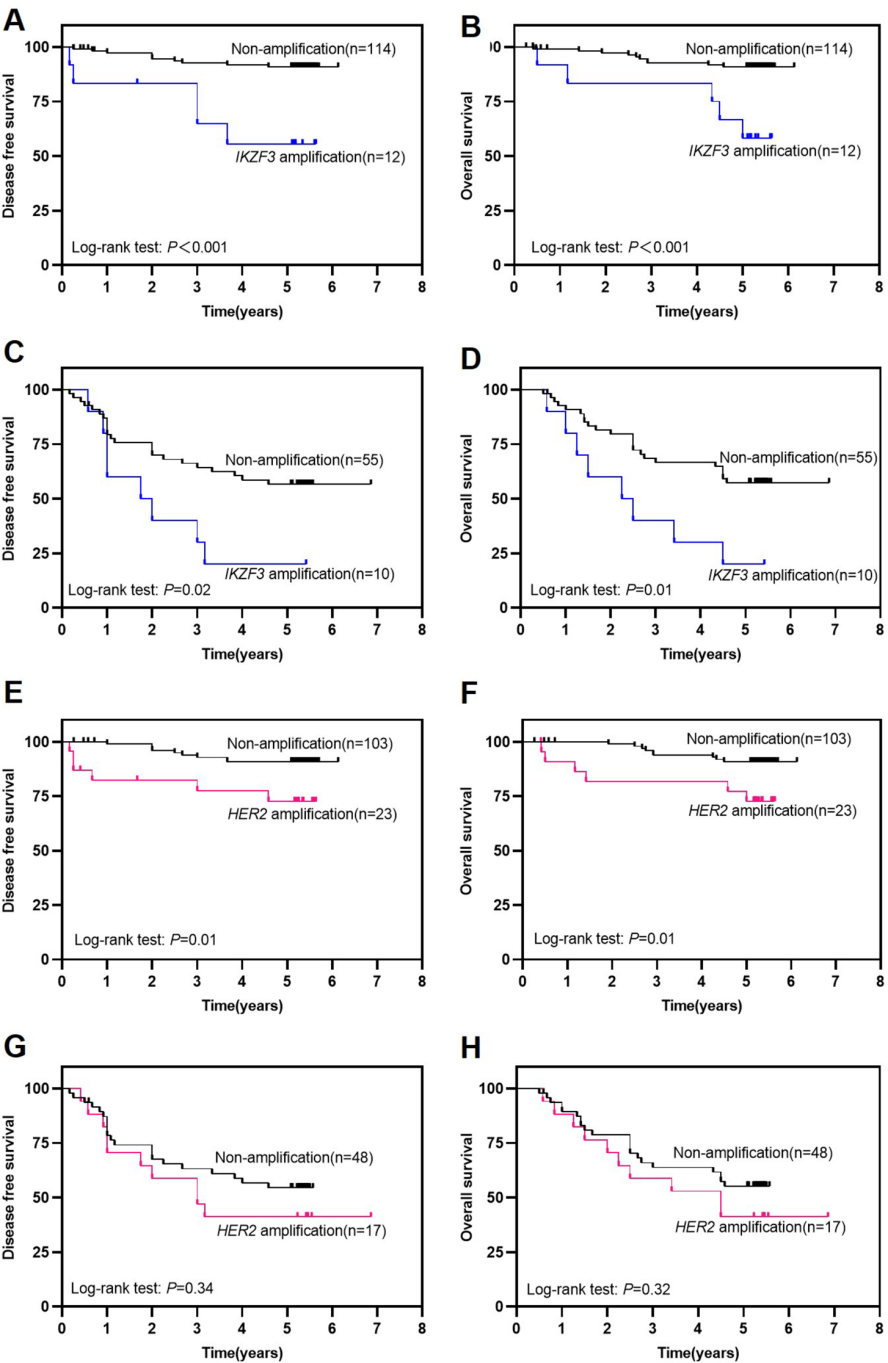
*IKZF3* amplification was related to unfavorable DFS and OS of IGC patients in stages I-II **(A-B)** and III-IV **(C-D)**. *HER2* amplification was associated with poorer DFS and OS in stages I-II IGC patients **(E-F)**, but not in stages III-IV **(G-H)**

Furthermore, in stages I–II IGC patients (*n* = 126, Fig. 3A-B), *IKZF3* amplification was correlated with poor DFS (*P* < 0.001) and OS (*P* < 0.001) significantly. A worse prognosis was observed in 12 patients with *IKZF3* amplification, with a median DFS and OS being 43.8 and 52.8 months compared with 57.6 and 58.6 months for 114 patients without *IKZF3* amplification. In stages III–IV IGC patients (*n* = 65, Fig. 3C-D), *IKZF3* amplification also showed poor clinical outcomes, with the median DFS and OS were 29.1 and 33.4 months respectively in 10 patients with *IKZF3* amplification, whereas it was 44.1 and 48.4 months respectively for 55 patients without *IKZF3* amplification (*P* = 0.02 for DFS and *P* = 0.01 for OS). On the other hand, *HER2* amplification exhibit significantly worse DFS and OS in stages I–II (*n* = 126, Fig. 3E-F) IGC patients, however not in stages III–IV (*n* = 65, Fig. 3G-H). Besides, in stages I–II IGC patients, univariate analysis suggests that *HER2* amplification, LN metastasis, *IKZF3* amplification, tumor deposits, and nerve invasion were significantly associated with DFS and OS (Supplemental information (SI) Table 1). In the multivariate analysis, *IKZF3* amplification (*P* = 0.019, HR: 3.737 for DFS; *P* = 0.036, HR: 3.203 for OS) and LN metastasis (*P* = 0.013, HR: 3.728 for DFS; *P* = 0.018, HR: 3.345 for OS) were associated with DFS and OS (SI Table 1). In stages III-IV IGC patients, univariate analysis suggests that age, *IKZF3* amplification, and tumor deposits exhibit correlation with both DFS and OS (SI Table 1). In the multivariate analysis, *IKZF3* amplification (*P* = 0.045, HR: 2.320 for DFS; *P* = 0.865, HR: 0.881 for OS) and tumor deposits (*P* = 0.007, HR: 1.286 for DFS; *P* = 0.26, HR: 1.25 for OS) were associated with DFS, but not with OS (SI Table 1). These data demonstrate that *IKZF3* amplification has independent prognostic significance for survival outcomes in IGC patients, regardless of patients in stages I-II or III-IV. *HER2* amplification is linked to poor prognosis in stages I-II IGC patients, but it was not identified as an independent prognostic factor (Table 2 and SI Table 1).

**Fig. 4:**
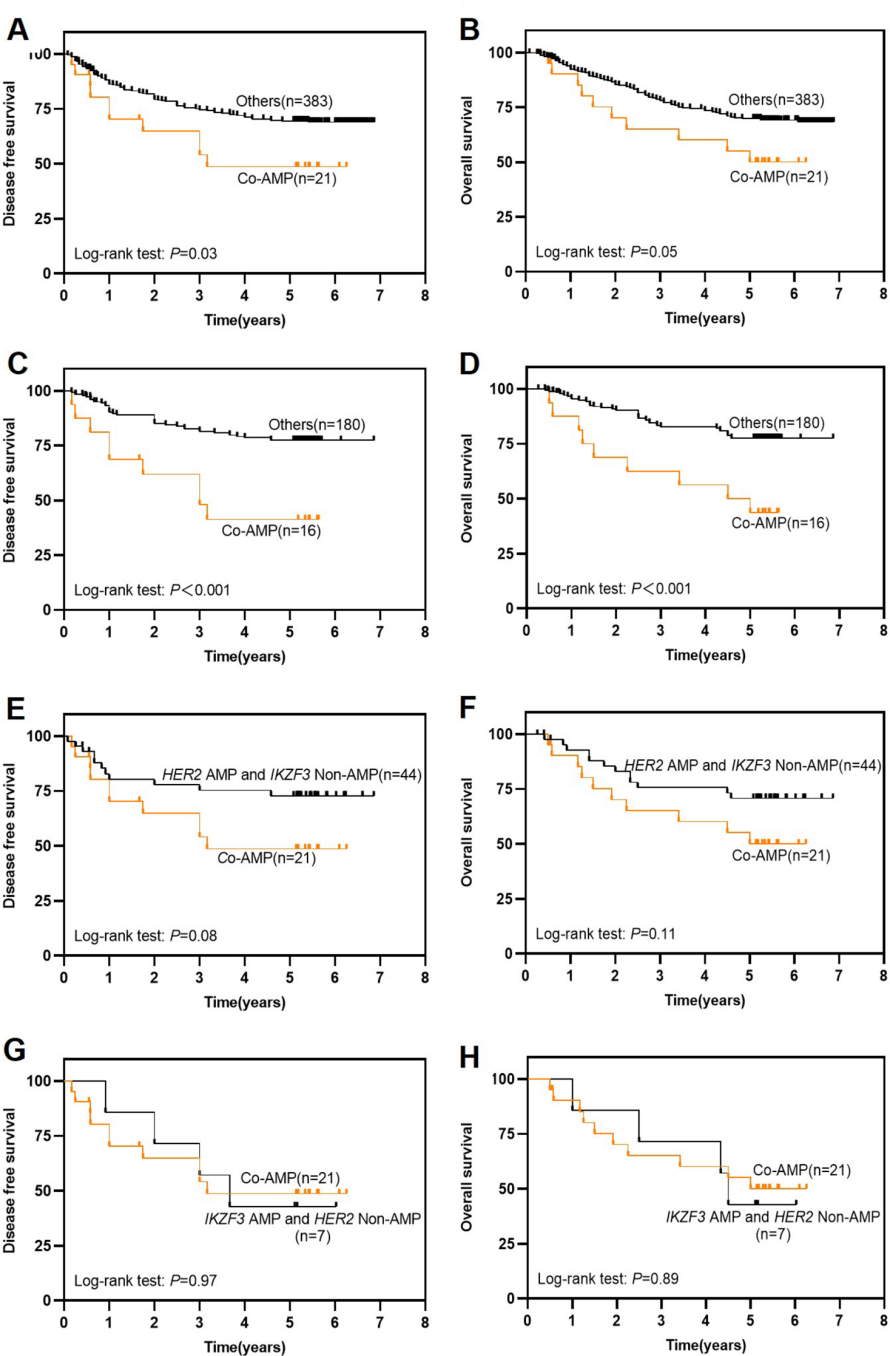
**(A-D)***IKZF3* and *HER2* co-amplification was associated with poorer DFS and OS for 404 patients **(A-B)** and 196 IGC patients **(C-D)**. **(E-F) ***IKZF3* amplification *in HER2-*amplified GC patients was related to potential poorer DFS and OS. **(G-H) ***HER2* amplification *in IKZF3-*amplified GC patients was not related to poorer DFS and OS

Ultimately, we conducted a comprehensive analysis of the prognosis in GC patients with co-amplification of *IKZF3* and *HER2*, and significant reductions were observed in both DFS and OS among all 404 GC patients (Fig. 4A-B) as well as IGC patients (Fig. 4C-D). Moreover, within the subset of patients with *HER2* amplification, those also exhibiting *IKZF3* amplification displayed a notably poorer prognosis (Fig. 4E-F). These findings suggest the additional clinical significance of *IKZF3* amplification in stratifying *HER2* amplified GC patients. However, it is worth noting that among patients with *IKZF3* amplification, there was no discernible impact on patient prognosis based on their *HER2* gene status (Fig. 4G-H).

## Discussion

Clinical treatment and prognosis of individuals diagnosed with GC are influenced by various factors, including pathological diagnosis, clinical staging, molecular pathological classification, etc. In most cases, patients with the same histologic type might display distinct molecular phenotypes and prognosis. It’s generally known that many non-anatomic factors, such as genetic and protein markers related to carcinogenesis and tumor invasion, have also been confirmed to have significant clinical implications for prognosis (Patel 2020). Therefore, accurately identifying new biomarkers in GC from multiple perspectives holds great prognostic significance and is more conducive to personalized therapy for GC patients after surgical treatment.

Many common amplifications in gastric cancer remain poorly elucidated, which could be crucial for us to understand the occurrence, development, invasion, as well as diagnosis and prognosis of GC. In this study, we attempted to explore common gene amplification events which might be of clinical significance for GC patients. We queried stomach cancer databases (TCGA, Nature 2014) deposited in cBioPortal, and the frequency of all amplified genes was ranked. An arbitrary cut-off of 10% frequency was used for gene amplification, *IKZF3* was selected for further analysis in 14 genes. *IKZF3* is a hematopoietic specific transcription factor that plays a pivotal role in the differentiation, proliferation, and development of both B and T lymphocytes (Li et al. 2018). In the field of solid tumours, bioinformatics-based research has demonstrated that *IKZF3* serves as an independent prognostic factor for both skin melanoma (SKCM) and human head and neck squamous cell carcinoma (HNSCC) (Li et al. 2023; Yang et al. 2022), suggesting its potential as a promising target for immunotherapy in the treatment of SKCM and HNSCC. However, the research on the association between *IKZF3* amplification and GC is scarce. In our study, *IKZF3* was found to amplified in 6.9% of GC patients, and it served as an independent prognostic marker indicating unfavorable outcomes, especially in IGC. The prognostic value of *IKZF3* was superior to *HER2* for GC patients.

In our study, patients with or without *IKZF3* amplification account for 6.9% (*n* = 28) and 93.1% (*n* = 376) respectively of all the 404 GC patients. We were surprised that *IKZF3* amplification showed a significant positive correlation with intestinal type of Lauren classification and *HER2* amplification (Table 1). Moreover, *IKZF3* amplification is significantly associated with vessel invasion in early gastric cancer, this indicate *IKZF3* amplification could potentially be a clue to distinguish between endoscopic submucosal dissection (ESD) and gastrectomy cases. Notably, IGC accounts for 78.6% (22/28) of GC patients with *IKZF3* amplification, and 63.1% (41/65) of GC patients with *HER2* amplification. In previous studies, HER2 expression had been observed to be higher in IGC compared to other types (Cruz-Reyes et al. 2013; Joshi et al. 2021), which is consistent to our results. On the other hand, *HER2* amplification accounts for 75.0% (21/28) in GC with *IKZF3* amplification. And conversely, *IKZF3* non-amplification group were about 88% *HER2* negative. This result aligns with the findings of preceding investigations (Lin et al. 2022), a bioinformatics-based study which involved these two genes in breast cancer, at a co-amplification rate of about 12–15%. This might be the result of that *IKZF3* is located in close proximity to the proposed ERBB2 17q12-q21 amplicon. This implies that *IKZF3* amplification in GC has certain differential diagnostic value.

As is well known, intestinal type (tubular and papillary) adenocarcinoma has a more favorable prognosis (Chen et al. 2015). Commonly associated with atrophic gastritis and gastric epithelial dysplasia, may express HER2 or PD-L1, both of which offer additional treatment options. Other types of GC, for instance diffuse type gastric carcinoma, consists of signet ring cells that are poorly differentiated, present as scattered individual cells or clusters, which is related to aggressive tumor proliferation, peritoneal metastasis, chemo resistance, and poor prognosis. In IGC instead of in other types of GC, *IKZF3* amplification is related to dismal prognosis, and act as an independent prognostic indicator in IGC, irrespective of the pTNM stages being I-II or III-IV (Fig. 3A-D). On the other hand, it is interesting to note that compared to other types of GC, *HER2* amplification is significantly linked to an unfavorable outcome in IGC, exclusively in stages I-II, but not in full cohort (Figs. 1I-J and 3E-H). In light of the evidence, it can be reasonably inferred that *IKZF3* amplification even outperformed *HER2* in predicting patient prognosis to some extent. Besides, among patients with *HER2* amplification, those who also exhibit *IKZF3* amplification have a worse prognosis (Fig. 4E-F), highlighting the clinical significance of *IKZF3* amplification in stratifying *HER2*-amplified GC patients.

Bioinformatics-based analysis shows *HER2* amplification, along with several co-amplified genes within a focal region, also known as the *HER2* amplicon, was determined to be a six-gene area including *PGAP3-ERBB2(HER2)-MIR4728-MIEN1-GRB7-IKZF3* (Li et al. 2020). In our current investigation, *HER2* amplification was observed in approximately 75% of cases with *IKZF3* amplification, which suggests the intriguing possibility that in addition to synergistically amplified with *HER2*, *IKZF3* amplification can also operate as an independent amplicon.

Generally, *HER2* is amplified in approximately 10–20% of gastric cancers (Boku 2014). Although 30 years have passed since the initial studies revealing a correlation between a positive *HER2* status and unfavorable prognosis were published, there remains ongoing debate surrounding this matter (Heike Grabsch 2010; Kurokawa et al. 2014; Lei et al. 2017; Wang et al. 2017). This may due to inadequate sample size, variations in population demographics, tumor heterogeneity, and discrepancies in assessment methods utilized across various studies, including antibody clones, evaluation criteria, and cut-offs applied for defining *HER2* positivity. Nevertheless, GC patients with *HER2* amplification gained improved overall survival on first-line trastuzumab–based therapy in recent years (Boku 2014; Joshi et al. 2021). However, approximately 30–60% patients experiencing disease progression after treatment with trastuzumab-based therapies exhibit *HER2* downregulation (Kanayama et al. 2018; Kim 2024; Sampera et al. 2019), it may result from either high percentage of primary and acquired resistance, which involves deregulation of PI3K/Akt pathway, *HER2* loss and heterogeneity of *HER2* proteome, or co-amplifications of *RTK/KRAS*, MET, CCNE1 (Joshi et al. 2021; Röcken 2022; Zhu et al. 2021). Our current research findings indicate simultaneous assessment of *HER2* and *IKZF3* gene status is indispensable for targeted therapy in GC, this can further stratify the risk of GC patients, evaluate patient prognosis and guide subsequent medication plans. Moreover, this may holds promise in improving clinical outcomes for patients with *IKZF3*/*HER2* co-amplification, which constitutes the main focus of our study. Based on our current results, screening drugs that target *IKZF3* amplification or *IKZF3*/*HER2* co-amplification, which can be developed and introduced into clinical practice is of important significance to GC patients, this part is currently in progress. This is also our main research direction in the future.

Nevertheless, our research has certain limitations. First, the use of TMA technique can underestimate or overestimate *IKZF3* status due to tumor and microenvironment heterogeneity. Second, in this study, the amplification cut-off for *IKZF3* was aligned with that of *HER2*, which was determined based on the efficacy of the targeted drug. Further research is needed to establish the optimal *IKZF3* amplification cut-off value for prognostic prediction. Third, IKZF3 protein level could not reflect *IKZF3* amplification status in our research. This is a very interesting point, the potential mechanisms including single nucleotide polymorphism in regulatory regions, post-transcriptional and post-translational modifications, mutations affecting upstream signaling pathways, as well as mutations that regulate protein stability. Further experiments are required to clarify this inconsistent mechanism.

This article is the first to elucidate the value of *IKZF3* amplification as a novel prognostic factor in GC patients, especially in IGC. Accordingly, we believe it is essential to trace the upstream and downstream signaling pathways associated with *IKZF3* to gain a comprehensive understanding of its role in the occurrence and development of GC as well as its implications in patient prognosis. In vitro and in vivo laboratory experiments are warranted in future.

## Conclusion

To summarize, our study revealed that *IKZF3* amplification is an independent prognostic factor in GC, which is superior to *HER2* to some extent. The prognostic significance of *IKZF3* requires more test-proof evidences in the future, which offers perspective on a potential role of *IKZF3* as a promising druggable target for GC as well as other carcinomas.

### Electronic supplementary material

Below is the link to the electronic supplementary material.

Supplementary Material 1

Supplementary Material 2

## Data Availability

No datasets were generated or analysed during the current study.
