# Supplementary figures and images for: IKZF3 amplification predicts worse prognosis especially in intestinal-type gastric cancer
Source: J Cancer Res Clin Oncol. 2024 Jul 25;150(7):363. doi: 10.1007/s00432-024-05868-2 (PMC11272681; doi:10.1007/s00432-024-05868-2)

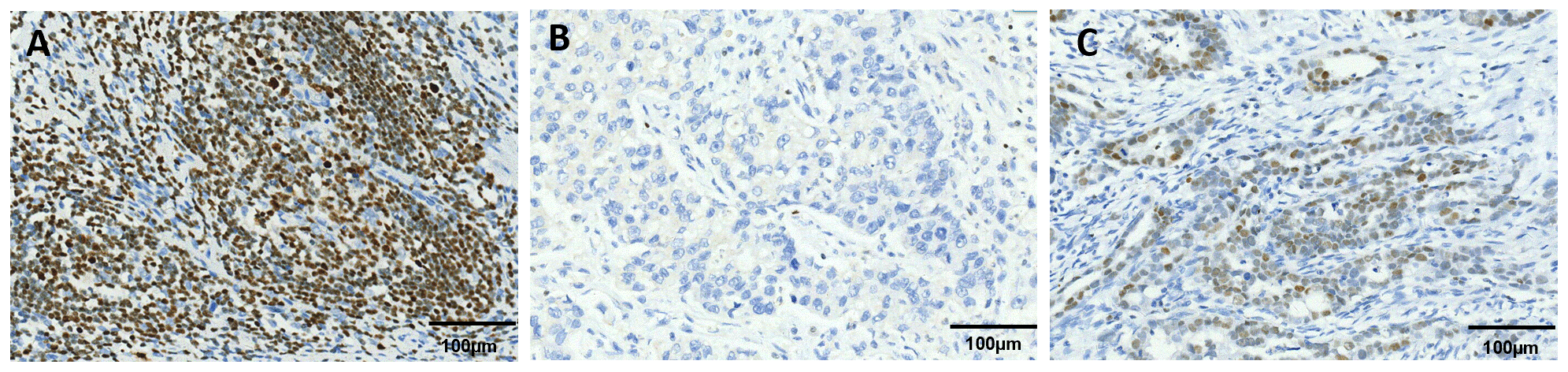

Supplement: Supplementary file 1 — Supplementary Material 1 [file 432_2024_5868_MOESM1_ESM.gif]
