# Supplementary material for: IKZF3 amplification predicts worse prognosis especially in intestinal-type gastric cancer
Source: J Cancer Res Clin Oncol. 2024 Jul 25;150(7):363. doi: 10.1007/s00432-024-05868-2 (PMC11272681; doi:10.1007/s00432-024-05868-2)
Supplement: Supplementary file 2 — Supplementary Material 2 [file 432_2024_5868_MOESM2_ESM.docx]

Supplemental information

Table 1 Univariate and multivariate survival analysis for OS and DFS in IGC (I-II and III-IV stages) patients.

| Variable | DFS( I-II)  HR  *P* value | | OS( I-II)  HR *P* value | | DFS(III-IV)  HR  *P* value | | OS(III-IV)  HR  *P* value | |
| --- | --- | --- | --- | --- | --- | --- | --- | --- |
| **Univariate analysis** |  |  |  |  |  |  |  |  |
| Sex | 0.243 | 0.170 | 0.234 | 0.166 | 0.396 | 0.127 | 0.425 | 0.158 |
| Age(years) | 0.597 | 0.417 | 0.571 | 0.379 | 0.424 | 0.004 | 0.392 | 0.030 |
| ***HER2* amp^★^** | 3.896 | 0.006 | 3.680 | 0.008 | 1.339 | 0.445 | 1.331 | 0.455 |
| **LN metastasis** | 4.647 | 0.002 | 4.429 | 0.002 | 1.497 | 0.581 | 1.924 | 0.371 |
| ***IKZF3* amp** | 6.160 | ＜0.001 | 5.785 | ＜0.001 | 2.355 | 0.038 | 2.707 | 0.016 |
| EBV infection | 0 | 0.999 | 0 | 0.999 | 0 | 0.999 | 0 | 0.999 |
| **Tumor deposits** | 1.920 | 0.003 | 1.889 | 0.005 | 1.263 | 0.007 | 1.219 | 0.018 |
| Vessel invasion | 2.438 | 0.079 | 2.490 | 0.073 | 1.301 | 0.458 | 1.289 | 0.475 |
| **Nerve invasion** | 5.305 | ＜0.001 | 5.452 | ＜0.001 | 0.917 | 0.847 | 00.947 | 0.882 |
| **Multivariate analysis** |  |  |  |  |  |  |  |  |
| Age |  |  |  |  | 0.467 | 0.078 | 0.640 | 0.242 |
| *HER2* amp | 2.312 | 0.126 | 1.958 | 0.236 |  |  |  |  |
| **LN metastasis** | 3.728 | 0.013 | 3.345 | 0.018 |  |  |  |  |
| ***IKZF3* amp** | 3.737 | 0.019 | 3.203 | 0.036 | 2.320 | 0.045 | 0.881 | 0.865 |
| **Tumor deposits** | 1.135 | 0.617 | 1.139 | 0.620 | 1.286 | 0.007 | 1.254 | 0.260 |
| Nerve invasion | 2.614 | 0.065 | 3.038 | 0.032 |  |  |  |  |

**^★^**Amp: amplification.
